# Exploring burnout and depression of Thai medical students: the psychometric properties of the Maslach Burnout Inventory

**DOI:** 10.5116/ijme.5dc6.8228

**Published:** 2019-11-29

**Authors:** Pongtong Puranitee, Siam Saetang, Sutida Sumrithe, Jamiu O. Busari, Walther N.K.A. van Mook, Sylvia Heeneman

**Affiliations:** 1 Ramathibodi Hospital, Faculty of Medicine, Mahidol University, Bangkok, Thailand; 2 Department of Educational Development & Research, Faculty of Health, Medicine & Life Sciences (FHML), Maastricht University (UM), The Netherlands; 3 School of Health Profession Education, Maastricht University (UM), Department of Intensive Care Medicine, The Netherlands; 4 Department of Pathology, Faculty of Health, Medicine & Life Sciences (FHML), School of Health Profession Education, Maastricht University (UM), The Netherlands

**Keywords:** Burnout, undergraduate medical student, medical education, medical student wellness, depression

## Abstract

**Objectives:**

To examine the psychometric properties of the Maslach Burnout Inventory-Student Survey (MBI-SS) Thai version and to determine the frequency of burnout and correlation between burnout and associated factors.

**Methods:**

A cross-sectional study was conducted among undergraduate medical students using convenience sampling (n=545, 76.1% response rate, female 52.1%). Data were collected by a self-report survey. The MBI-SS was translated in Thai and tested for internal consistency using Cronbach’s coefficient alpha. A confirmatory factor analysis was performed using as fit indices of the chi-square and degree of freedom ratio (χ^2^/df), Comparative Fit Index (CFI), the Goodness of Fit Index (GFI), the Non-normed Fit Index (NNFI), Akaike information criterion (AIC) and the Root Mean Square Error of Approximation (RMSEA). Spearman and Kendall’s tau-b were used to identify correlations between burnout, depression and other factors.

**Results:**

Interrater reliability was acceptable with Kappa of 0.83. Confirmatory factor analysis demonstrated good fit indices (χ^2^/df=197.62/83, CFI=0.97, GFI=0.95, NNFI=0.96, AIC=271.62 and RMSEA=0.06). Burnout had a weak, positive association with the PHQ-9 (r=0.294, df=2, p< 0.001). The screening depression score had a significant, modest positive association with emotional exhaustion (r=0.469, df=4, p<0.001) and cynicism (r=0.411, df=4, p< 0.001), and a weak inverse association with professional efficacy (r=−0.273, df=4, p< 0.001).

**Conclusions:**

The Thai version of the MBI-SS had adequate psychometric properties among Thai medical students and can be used to assess burnout among undergraduate medical students in Thailand. Burnout was associated with risk for depression. Further studies on other associated factors contributing to depression are suggested.

## Introduction

Burnout, as defined by Maslach^1^, includes three key subscales, namely, high emotional exhaustion, high depersonalisation and lack of professional efficacy, which relates to the critical social environment in the health care setting. Emotional exhaustion is one of the responses of stress when people feel overwhelmed by job demands but lack emotional or physical resources to cope with these demands. Depersonalisation represents the interpersonal context component that refers to a negative, hard-hearted or excessively detached response to various aspects of the job. Lack of professional efficacy is a feeling of self-incompetence, lack of reducibility or lack of job achievement.^2^ The Maslach Burnout Inventory (MBI) is a self-assessment questionnaire that was introduced in 1981^3^ and is the gold standard for measuring burnout.^3^ The MBI has three versions: Human Services survey (MBI-HSS), General survey (MBI-GS) and General survey for student (MBI-GS(S)) and Educators survey (MBI-ES). In 2002, Schaufeli and colleagues^4^ developed the Maslach Burnout Inventory – Student Survey (MBI-SS) to assess burnout among university students and consisting of 15 items rated by a seven-point frequency rating scale ranging from 0 (never) to 6 (always) and three subscales: emotional exhaustion (five items), depersonalisation (four items) and professional efficacy (six items).

The psychometric properties of the MBI-SS were shown to have adequate validity and reliability in Spain, Portugal, The Netherlands, Brazil, Italy and France.^4^^-^^7^ Among non-Western countries, the MBI-SS is found to have acceptable psychometric properties in Serbia, Turkey (high school students), Iran and South Korea.^8^^-^^12^ However, the Japanese version of the MBI-SS needed minor changes to improve the fit of its three-factor model.^13^ In addition, Hu and Schaufeli^14^ demonstrated low internal consistency of the Chinese MBI-SS necessitating the reformulation of some items. In summary, the MBI-SS of some non-Western countries needed some adaptation of items to improve its validity and reliability. This may also apply to Thailand. The Thai culture may contrast with most Western countries and even with the culture in some non-Western countries. Furthermore, studies in Thailand have so far focused on residents, not on undergraduate medical students. Moreover, a Thai version of the MBI-SS is lacking and the validity of its psychometric properties has not been studied so far. Therefore, the construction of the Thai version of the MBI-SS and subsequent testing of its psychometric properties among Thai medical students before utilisation are needed.Consequently, this study aimed to test the psychometric properties of the Thai version of the Maslach Burnout Inventory-Student Survey (MBI-SS) and to determine if the occurrence of burnout and its subscales are potentially correlated with depression, years of training, gender and grade point average (GPA) among Thai undergraduate medical students.

A Thai version of the MBI-SS with sufficient psychometric properties would guarantee the accurate measurement of burnout among undergraduate medical students and improve understanding of burnout in medical students in non-Western countries.

## Methods

### Participants

A cross-sectional study was conducted among undergraduate medical students in Ramathibodi Hospital, Mahidol University, Bangkok, Thailand, using convenience sampling (*n*=545, 76.1% response rate, female 52.1%). Participants who incompletely responded and those with extreme responses to multiple questions in the questionnaire were excluded. Four hundred fifty-one completed responses (62.9%) were finally included. In the preclinical years, 361 medical students completed the survey (48.3% female). In the clinical years, 190 medical students completed the surveys (52.1% female). Medical students in the preclinical and clinical years were asked to complete the written questionnaire in the last semester of the academic year 2016. The curriculum consisted of a three-year preclinical course (mostly classroom learning) and three-year clinical clerkships. Year 2–5 undergraduate medical students were enrolled to represent both preclinical and clinical years. First- and sixth-year medical students studying outside Ramathibodi Hospital were excluded. All participants provided written informed consent prior to responding and subsequent data collection was confidential. Data handling and accessibility were allowed only for the members of the research team. This study was approved by the Ethics Committee of the Faculty of Medicine of Ramathibodi Hospital, Mahidol University.

### Procedure

Permission to translate the Maslach Burnout Inventory-general survey for students into a Thai version was obtained from Mind Garden, Inc. The Thai version of the Maslach Burnout Inventory-general survey for students (MBI-SS) was developed to measure burnout among Thai undergraduate medical students by a two-stage process, namely, the translation process and testing the resulting version for factorial validity and psychometric properties. The translation process—‘forward’ translation from English to Thai and ‘backward’ into English—was done by two independent experienced translators. The Thai translation was reviewed by a panel of experts including three faculty medical instructors (from paediatrics, family medicine, and clinical epidemiology departments) and based on their suggestions, minor cultural adaptations to fit with Thai context, and to maintain translation quality was performed. The expert panel and a native English speaker (a physician from the family medicine department) then rated for degree of agreement with the original version and the back-translation into English. Finally, pilot testing was performed that showed 10 medical students had no difficulties in understanding and completing the questionnaire.

The next phase consisted of psychometric properties testing of the Thai version of the MBI-SS, 15- item questionnaire consisted 3 subscales: Emotional exhaustion (5 items), Depersonalization (4 items), Professional efficacy (6 items) and rated by 7-point frequency rating scale ranging from 0 (never) to 6 (always). Emotional exhaustion scores over 14 are considered as high level, depersonalization scores over 6 are considered as high level, and personal efficacy score lower than 23 is considered as low level. A study in Spanish (n = 621), Portuguese (n = 723), and Dutch (n = 309) revealed that the three-factor structure (emotional exhaustion, depersonalization, and personal efficacy) of the MBI-SS fits to the data  in the Spanish (χ^2^/df=209.48/81, CFI=0.95, TLI=0.94 and RMSEA=0.05), Portuguese (χ^2^/df=280/82, CFI=0.93, TLI=0.92 and RMSEA=0.06), and Dutch (χ^2^/df=127.14/83, CFI=0.97, TLI=0.96 and RMSEA=0.04). Intercorrelation and internal consistencies (Cronbach’s alpha values) of the MBI-SS subscales of Spain, Portugal, Netherland were 0.74-0.79, 0.69-0.86, and 0.67-0.86, respectively. Most of them met the criterion of 0.7.^4^

Data collected included gender, study year, grade point average (GPA), non-academic time, sleeping time and screening scores for students’ depression (see below). The GPA refers to the cumulative average of grades during their course of study and reflects academic performance on a scale of 0–4 (4 is the best academic performance score). A GPA of less than two is considered to be a ‘fail’ in terms of summative assessment, and the student would then be expected to repeat certain blocks or subjects in the next academic year.

The depression screening tool used in this study is the Thai version of the Patient Health Questionnaire-9 (PHQ-9), a 9-item validated questionnaire. The Thai version of the PHQ-9 had satisfactory internal consistency (Cronbach’s alpha = 0.79) and has acceptable psychometric properties for screening for major depression in general practice with cut-off score of greater than 9.^15^

The 33-item written questionnaire including demographic data was subsequently distributed in written format among pre-clinical and clinical year medical students after lecture hall activities by the first author in the last semester of the academic year in February to June 2017, a strategy empirically known to provide a higher response rate in Thai culture.

### Data analysis

The interrater reliability (IRR) by means of Kappa was assessed to identify the degree of agreement. Data from the questionnaire was tested for psychometric properties. Internal consistency was analysed to demonstrate the level of reliability, measured with a standardised Cronbach’s coefficient alpha. A Cronbach’s alpha ≥ 0.7 was considered acceptable, although we preferred alpha ≥ 0.8.^16^

As cross-culture and language differences between Western and non-Western countries have to be taken into account, a confirmatory factor analysis (Principal Component Analysis) was used to confirm the structure validity of the Thai version MBI-SS and to compare similarity to the original hypothesised measurement model MBI-SS, English version. First, the psychometric sensitivity evaluation was conducted by measuring central tendency and shape. Items with skew (Sk) above three and kurtosis (ku) above 3, in absolute values, were directed to sensitivity problems. Fit indices used for confirmatory factor analysis were the chi-square and degree of freedom ratio (χ^2^/df), Comparative Fit Index (CFI), the Goodness of Fit Index (GFI), the Non-normed Fit Index (NNFI), Akaike information criterion (AIC) and the Root Mean Square Error of Approximation (RMSEA). The model fit was considered suitable for χ^2^/df (chi-square and degree of freedom ratio) values of less than 5. When the CFI, GFI, and NNFI values were greater than 0.90, the model was considered an adequate fit.^4^^,^^17^^,^^18^ A RMSEA value of less than 0.08 indicated an acceptable model-data fit, whereas a value above 0.10 indicated that the model should be rejected.^4^^,^^19^Spearman and Kendall’s tau-b method was used to identify correlations between burnout and other factors. Confirmatory factor analysis, descriptive data, and correlation analysis was performed by using AMOS® version18.0 program, SPSS Statistics for Windows, version x.0 (SPSS Inc., Chicago, IL, USA).

## Results

### Reliability

In the translation process from the English to the Thai version, the backward translation and the original English version were compared and analysed for interrater reliability. The Thai version of MBI-SS showed an acceptable value of Kappa = 0.83.

The internal consistency of the questionnaire was good with a Cronbach’s alpha value of 0.80. The Cronbach’s alpha coefficient values for emotional exhaustion, depersonalisation and person accomplishment were 0.89, 0.81, and 0.70, respectively. Mean, standard deviation and intercorrelations between each subscale are reported in Table 1.

**Table 1:** Means, standard deviations, Cronbach’s indices of internal consistency and intercorrelations

Burnout Subscales	Mean	SD	Cronbach’s alpha	Intercorrelations	
Emotion exhaustion	16.20	6.90	0.8888		
Depersonalization	9.08	5.44	0.8124	0.6002	
Personal efficacy	20.85	5.93	0.6979	0.1616	-0.0192

### Confirmatory factor analysis

The CFI and GFI values of the three-factor model (M1) were 0.85 and 0.85, which did not satisfy the respective criteria. The modified three-factor model (M2) was slightly adjusted using modification indices provided by AMOS. The modified three-factor model (M2) improved the model–data fit to acceptable values of CFI=0.97 and GFI= 0.95. The confirmatory factor analysis demonstrated good fit indices of the Thai version of the MBI-SS, namely, X^2^/df=197.62/83, CFI=0.97, GFI=0.95, NNFI=0.96, AIC=271.62, and RMSEA=0.06 (Table 2).

**Table 2:** Results of the confirmatory factor analysis of the Thai version of MBI-SS

Acceptable values^4,17–19^	Chi-square	Degree of freedom	CFI	GFI	NNFI	AIC	RMSEA
	Chi-square/degree of freedom <5		>0.90			-	<0.08
Model 1 (M1): Three factors	598.28	87	0.85	0.86	0.82	664.38	0.11
Model 2 (M2): Modified three factors	197.62	83	0.97	0.95	0.96	271.62	0.06

**Figure 1:**
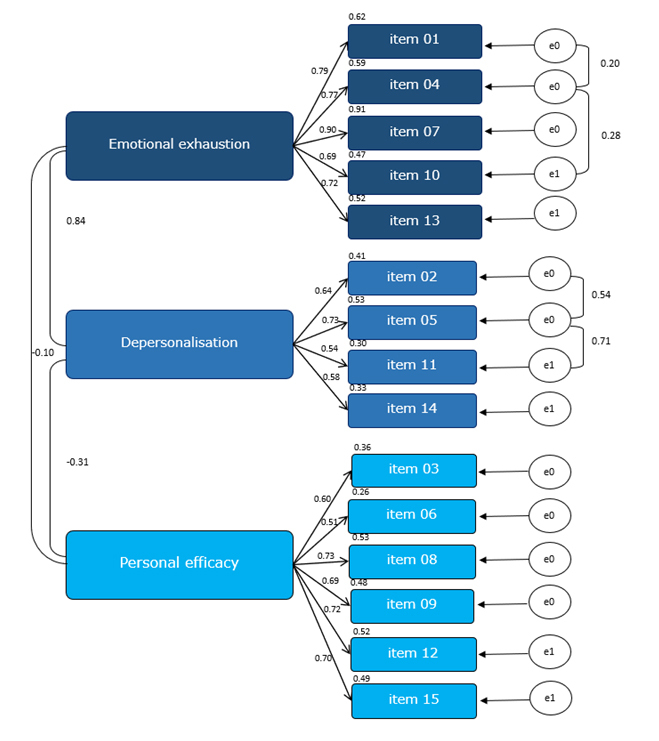
Path diagram with standard factor loadings of the 15-item Thai version of MBI-SS (n=451)

The path diagram with standard factor loadings of the 15-item Thai version of MBI-SS (Figure 1) indicated standardised coefficients of the relationship between factors and items between 0.51–0.90 (Table 3).

**Table 3:** Standardised coefficients of the relationship between factors and items of the Thai version of MBI-SS (n=451)

Item	Coefficients	Standard Error	Factor Loading
Emotional Exhaustion			
item 1	1		0.790
item 4	0.988	0.051	0.770
item 7	1.121	0.056	0.897
item 10	0.921	0.062	0.685
item 13	1.016	0.064	0.719
Depersonalisation			
item 2	1		0.641
item 5	1.055	0.061	0.730
item 11	0.759	0.084	0.544
item 14	0.81	0.085	0.575
Personal Efficacy			
item 3	1		0.603
item 6	0.918	0.103	0.513
item 8	1.316	0.114	0.729
item 9	1.116	0.1	0.692
item 12	1.228	0.107	0.719
item 15	1.127	0.1	0.700

The average GPA was 3.27 and 3.22 among pre-clinical and clinical year medical students, respectively (Table 4). Forty percent of the participants had a high risk of depression (score above 9) as identified by the PHQ-9 screening tool. The prevalence of burnout on all three subscales among medical students was 28.4% (n=128) as identified both by low personal accomplishment, high emotional exhaustion, and high depersonalisation. Medical students’ perceptions of burnout on subscales were 54.8% for low personal accomplishment, 57.4% for high emotional exhaustion and 65% for high depersonalisation.

### Associated factors

Statistically significant, yet weak negative correlations between burnout and GPA (*r* =−0.119, df=3, *p*=0.002) and gender (male) (*r* =−0.139, df=1, *p*=0.003) were found. Students who had better academic performance and male gender had lower risk for burnout. Burnout also had weak positive associations with the depression score (PHQ-9, *r*=0.294, df=2, *p*<0.001). Risk of depression had a significant, yet modest positive association with emotional exhaustion (*r*=0.469, df=4, *p*<0.001) and depersonalisation (*r*=0.411, df=4, *p*<0.001), and a weak inverse association with professional efficacy (*r*=−0.273, df=4, *p*<0.001). These findings indicate that students that experienced emotional exhaustion and/or depersonalisation were at risk for depression as well as students who perceived low personal efficacy. Female gender was positively associated with professional efficacy (*r*=0.104, df=2, *p*=0.020). A higher GPA was weakly and negatively associated with depersonalisation (*r*=-0.112, df=6, *p*=0.017) (Table 5).

**Table 4:** Baseline characteristics of the participants (N=451)

Baseline Characteristics		n	%
Year			
	· 2^nd^	125	27.7
	· 3^rd^	136	30.2
	· 4^th^	143	31.7
	· 5^th^	47	10.4
GPA			
	· <2.80	63	14.0
	· 2.80–3.249	149	33.0
	· 3.25–3.499	107	23.7
	· ≥3.5	132	29.3
Mean (SD)		3.25	(0.37)
Min (max)		2.11	(3.97)
Gender		n	%
	· Male	225	49.9
	· Female	226	50.1
PHQ-9 Depression score			
	· 0-4	115	25.5
	· 5-9	153	33.9
	· 10-14	140	31.0
	· 15-19	29	6.4
	· 20-27	14	3.1
Burnout subscale			
Personal efficacy			
	High	85	18.8
	Moderate	119	26.4
	Low	247	54.8
Emotional exhaustion			
	Low	83	18.4
	Moderate	109	24.2
	High	259	57.4
Depersonalisation			
	Low	30	6.7
	Moderate	128	28.4
	High	293	65.0

## Discussion

This study investigated burnout among Thai undergraduate medical students. It tested the psychometric properties of the Thai version of the Maslach Burnout Inventory-Student Survey (MBI-SS) and determined if the occurrence of burnout and its subscales were potentially correlated with other factors such as depression, years of training, gender and grade point average (GPA). The results revealed acceptable interrater reliability with Kappa of 0.83, and confirmatory factor analysis demonstrated good fit indices. Thus, this study revealed sufficient reliability and validity of the Thai version of the MBI-SS. The internal consistency with a Cronbach’s alpha value of 0.80 was found to be acceptable as also reported by previous studies in Spain, Portugal, Brazil, Turkey (high school students) and Iran,^4–7^ but somewhat contrasting to the results of a study on the Chinese MBI-SS.^14^ In the Chinese context, items 4 and 13 were ambiguous because the activities during a day in university or attending in the class were perceived to be pleasant or relaxing. 

**Table 5:** Correlations between burnout, its subscales and factors

Factors	Burnout			Professional Efficacy			Emotional Exhaustion			Depersonalisation		
	correlation	p-value	df	correlation	p-value	df	correlation	p-value	df	correlation	p-value	df
Year^†^	-0.055	0.203	3	0.083	0.652	6	0.063	0.131	6	0.029	0.501	6
GPA	-0.119^*^	0.002	3	0.234	<0.001	6	-0.053	0.265	6	-0.112^*^	0.017	6
Gender^‡^	-0.139^*^	0.003	1	0.104^*^	0.020	2	-0.064	0.153	2	0.059	0.200	2
Depression screening (PHQ9)	0.294^*^	<0.001	2	-0.273^*^	<0.001	4	0.469^*^	<0.001	4	0.411*	<0.001	4

^*^Statistically significant with p<0.005, ^†^Spearman correlation, ^‡^Kendall’s tau-b correlation, df = degree of freedom

Therefore, the suggestion was to reformulate these two items to make them more specific for studying.^14^ Similarly, the Japanese version of the MBI-SS was suggested to revise the word ‘tsukare’ in items1 and 2 to differentiate between the original English words of ‘drained’ and ‘used up’.^1^

In this study, the confirmatory factor analysis confirmed that the three-factor model in the translation fitted the initial structure and, thus, has sufficient validity without the need for reformulation of the items. This contrasts with the Japanese and Chinese versions in which some words needed to be reformulated due to the ambiguous meanings in their cultures. This suggests that languages and terminology of the seemingly same word could have different meaning and refer to different things in different countries with different context.

The prevalence of burnout (as identified by the three subscales of low personal accomplishment, high emotional exhaustion *and* high depersonalisation) was 28%. This prevalence is lower than reported in most of the previous studies among medical and health science students in which the burnout prevalence was up to 70%.^20^^-^^25^ However, the majority of the Thai undergraduate medical students perceived either low personal accomplishment, high emotional exhaustion *or *depersonalisation, which is similar to studies conducted internationally. ^20^^-^^24^ This study suggests that students of male gender and lower academic performance were at higher risk of burnout. Although Backović and colleagues ^25^ found that *female students* were more vulnerable to stress and burnout, Chunming and colleagues^26^ also indicated that *male students* had greater burnout risk.

In China, Liu and colleagues^27^ showed an association between higher grades and increased burnout risk, which was in contrast to this study. We speculate that in Thai culture, GPA may be important for professional identity formation. Medical students with higher GPA may feel more relieved after achieving these academic goals and may be become more popular and accepted among peers, teachers or even their families. Consequently, they might perceive a higher level of personal accomplishment and, thus, be at lower risk for burnout. Evidence of a correlation between burnout and depression was also demonstrated in this study: burnout had a weak, positive association with the PHQ-9. The screening depression score had a significant, but modest positive association with emotional exhaustion and cynicism, and a weak negative association with professional efficacy. Students who experience high emotional exhaustion and/or  depersonalisation as well as low personal efficacy were at risk for depression, similar to the findings of a previous study in Oman.^28^ The findings of the present study, when put in perspective of the literature, underscore that the demonstrated link between burnout and depression among medical students is to some extent generalizable across cultures. In addition, in a study of freshman and sophomore premedical students of Indiana University, Grace^29^ also indicated a positive relationship between depressive symptoms and burnout. This indicates that early signs of these conditions could pre-exist before entering medical education and, subsequently, might increase during the process of professional identity formation while in medical school.^30^ Moreover, burnout and depressive symptoms were found to be associated with suicidal risk.^31^ Similar to this study, Kroskaa and colleagues^32^ and Rotenstein and colleagues^33^ found 25% and 28%, respectively, of medical students, experience depressive symptoms. However, the depression screening tool used in this study was to identify high-risk students to be able to offer further formal mental health diagnosis and treatment. The results of this study could contribute to insight and raised awareness amongst medical teachers and educationalists of the potential contributory factors and impact of burnout among medical students.

### Strengths and limitations

The strengths of this study include the adequate sample size of the students that helped facilitate the factorial validation of the items and test of the psychometric properties of the questionnaire. This study also provides evidence that the psychometric properties of the Thai version of MBI-SS are valid and allow opportunities for further research on burnout within the Thai context. It is important to note that the prevalence of depression among medical students was not included in this study, and the PHQ-9 was used as a screening tool that only indicates risk of depression and not a diagnostic tool. A limitation of this study was the self-administered questionnaires, which might have been answered dishonestly, due to social desirability. Also, some students who were experiencing burnout or depression might not have responded, so the reported frequency might be an underestimate.

Figure 1. Path diagram with standard factor loadings of the 15-item Thai version of MBI-SS (n=451)

## Conclusions

This study provides new insight regarding the psychometric properties of this Thai version of the MBI-SS. It shows that the items have a good fit in the structure of the questionnaire and are valid without the need for any reformulation. The interrater reliability of the items and the internal consistency were sufficient. Thus, the Thai version of the MBI-SS can potentially be used to assess burnout among undergraduate medical students in Thailand both in the preclinical and clinical years. The prevalence of burnout among medical students was 28.4%. A correlation between burnout, its subscales, and risk for depression was identified for Thailand, similarly to other cultures. Male gender and lower academic performance (GPA) were associated with higher risk of burnout. Further studies on other associated factors contributing to burnout and risk for depression in non-Western context are needed to provide more insights and facilitate transfer to potential solutions to alleviate these interlinked problems.

### Acknowledgements

This research was funded by the Faculty of Medicine Ramathibodi Hospital, Mahidol University, Bangkok, Thailand. The authors would like to express gratitude to Assoc. Prof Dr Pornthep Tanpowpong for aspiring guidance, constructive feedback and Prof Dr Manote Lotrakul for giving permission of the Thai version of the Patient Health Questionnaire-9.

### Conflict of Interest

The authors declare that they have no conflict of interest.

## References

[1] Schaufeli WB, Maslach C, Marek T. Professional burnout: recent developments in theory and research. New York: CRC Press; 2018.

[2] Maslach C, Leiter MP (2016). Understanding the burnout experience: recent research and its implications for psychiatry.. World Psychiatry.

[3] Maslach C, Jackson SE (1981). The measurement of experienced burnout.. J Organiz Behav.

[4] Schaufeli WB, Martínez IM, Pinto AM, Salanova M, Bakker AB (2002). Burnout and engagement in university students.. Journal of Cross-Cultural Psychology.

[5] Campos JA, Maroco J (2012). [Maslach Burnout Inventory - Student Survey: Portugal-Brazil cross-cultural adaptation].. Rev Saude Publica.

[6] Portoghese I, Leiter MP, Maslach C, Galletta M, Porru F, D'Aloja E, Finco G, Campagna M (2018). Measuring burnout among university students: factorial validity, invariance, and latent profiles of the Italian version of the Maslach Burnout Inventory Student Survey (MBI-SS).. Front Psychol.

[7] Faye-Dumanget C, Carré J, Le Borgne M, Boudoukha PAH (2017). French validation of the Maslach Burnout Inventory-Student Survey (MBI-SS).. J Eval Clin Pract.

[8] Ilic M, Todorovic Z, Jovanovic M, Ilic I (2017). Burnout syndrome among medical students at one University in Serbia: validity and reliability of the Maslach Burnout Inventory - Student Survey.. Behav Med.

[9] Kutsal D, Bilge F. A study on the burnout and social support levels of high school students. Eğitim ve Bilim. 2012;37(164):283-297.

[10] Yavuz G, Dogan N (2014). Maslach Burnout Inventory-Student Survey (MBI-SS): a validity study.. Procedia - social and behavioral sciences.

[11] Rostami Z, Abedi MR, Schaufeli WB, Ahmadi SA, Sadeghi AH. The psychometric characteristics of Maslach Burnout Inventory Student Survey: a study students of Isfahan University. Zahedan Journal of Research in Medical Science. 2014; 16(9):55-58.

[12] Shin H, Puig A, Lee J, Lee JH, Lee SM (2011). Cultural validation of the Maslach Burnout Inventory for Korean students.. Asia Pacific Educ Rev.

[13] Tsubakita T, Shimazaki K (2016). Constructing the Japanese version of the Maslach Burnout Inventory-Student Survey: confirmatory factor analysis.. Jpn J Nurs Sci.

[14] Hu Q, Schaufeli WB (2009). The factorial validity of the Maslach Burnout Inventory - Student Survey in China.. Psychol Rep.

[15] Lotrakul M, Sumrithe S, Saipanish R (2008). Reliability and validity of the Thai version of the PHQ-9.. BMC Psychiatry.

[16] Kline P. Handbook of psychological testing. London: Taylor & Francis; 2013.

[17] Maroco J. Análise de Equações Estruturais: fundamentos teóricos, software and aplicações. Lisboa: Saraiva; 2010.

[18] Zhang Y, Gan Y, Cham H (2007). Perfectionism, academic burnout and engagement among Chinese college students: A structural equation modeling analysis.. Personality and Individual Differences.

[19] Hoyle RH. Structural equation modeling: concepts, issues, and applications. London: SAGE Publications; 1995.

[20] Almeida GC, Souza HR, Almeida PC, Almeida BC, Almeida GH (2016). The prevalence of burnout syndrome in medical students.. Arch Clin Psychiatry (São Paulo).

[21] Santen SA, Holt DB, Kemp JD, Hemphill RR (2010). Burnout in medical students: examining the prevalence and associated factors.. South Med J.

[22] Dyrbye LN, Thomas MR, Huntington JL, Lawson KL, Novotny PJ, Sloan JA, Shanafelt TD (2006). Personal life events and medical student burnout: a multicenter study.. Acad Med.

[23] IsHak W, Nikravesh R, Lederer S, Perry R, Ogunyemi D, Bernstein C (2013). Burnout in medical students: a systematic review.. Clin Teach.

[24] Boni RAS, Paiva CE, de Oliveira MA, Lucchetti G, Fregnani JHTG, Paiva BSR (2018). Burnout among medical students during the first years of undergraduate school: prevalence and associated factors.. PLoS ONE.

[25] Backović DV, Zivojinović JI, Maksimović J, Maksimović M. Gender differences in academic stress and burnout among medical students in final years of education. Psychiatr Danub. 2012;24(2):175-181. 22706416

[26] Chunming WM, Harrison R, MacIntyre R, Travaglia J, Balasooriya C (2017). Burnout in medical students: a systematic review of experiences in Chinese medical schools.. BMC Med Educ.

[27] Liu H, Yansane AI, Zhang Y, Fu H, Hong N, Kalenderian E (2018). Burnout and study engagement among medical students at Sun Yat-sen University, China.. Medicine (Baltimore).

[28] Al-Alawi M, Al-Sinawi H, Al-Qubtan A, Al-Lawati J, Al-Habsi A, Al-Shuraiqi M, Al-Adawi S, Panchatcharam SM (2019). Prevalence and determinants of burnout syndrome and depression among medical students at Sultan Qaboos University: a cross-sectional analytical study from Oman.. Arch Environ Occup Health.

[29] Grace MK (2018). Depressive symptoms, burnout, and declining medical career interest among undergraduate pre-medical students.. Int J Med Educ.

[30] Goel AD, Akarte SV, Agrawal SP, Yadav V (2016). Longitudinal assessment of depression, stress, and burnout in medical students.. J Neurosci Rural Pract.

[31] Dyrbye LN, Thomas MR, Massie FS, Power DV, Eacker A, Harper W, Durning S, Moutier C, Szydlo DW, Novotny PJ, Sloan JA, Shanafelt TD (2008). Burnout and suicidal ideation among U.S. medical students.. Ann Intern Med.

[32] Kroska EB, Calarge C, O’Hara MW, Deumic E, Dindo L (2017). Burnout and depression in medical students: relations with avoidance and disengagement.. Journal of Contextual Behavioral Science.

[33] Rotenstein LS, Ramos MA, Torre M, Segal JB, Peluso MJ, Guille C, Sen S, Mata DA (2016). Prevalence of depression, depressive symptoms, and suicidal ideation among medical students.. JAMA.

